# Supplementary material for: Factors Associated with Over-the-Counter Analgesic Overuse among Individuals Experiencing Headache
Source: Clin Pract. 2022 Sep 5;12(5):714–22. doi: 10.3390/clinpract12050074 (PMC9498430; doi:10.3390/clinpract12050074)
Supplement: Supplementary file 1 [file clinpract-12-00074-s001.zip › clinpract-1804387-supplementary.pdf]

Questionnaire :

1. Do you have headache
    a. Yes
    b. No

Demographic data:

2. Gender
    a. Male
    b. female
3. Nationality
    a. Saudi
    b. No Saudi
4. Do you work
    a. Yes
    b. No
5. Did you lose work recently?
    a. Yes
    b. No
6. Work sector:
    a. Public
    b. Private
    c. Self business
7. occupation
    a. education
    b. trading
    c. health care
    d. governmental
    e. military
8. do you work in more than one job?
    a. Yes
    b. No
9. How many hours do you work per day
    a. Less than 8 hours
    b. 8-12 hours
    c. More than 12 hours
10. income
    a. sufficient
    b. not sufficient
11. age
12. marital status
    a. single
    b. married
    c. divorce or widow
13. education
    a. high school
    b. bachelor degree
    c. post graduate

d. other

14. children:
    a. no children
    b. yes

Factors affect headache

15. history of chronic medical disease
    a. yes
    b. no
16. What is it(mention)?
17. sleeping hours
    a. less than 6 hours
    b. more than 7 hours

18. smoking :
    a. yes
    b. no
19. any caffeinated beverage do you use :
    a. yes
    b. no
20. ifyes how many cup?
    a. 1-2 cup
    b. 3 cups
    c. more than 4 cups

History of headache

Criteria for medication over use headache

21. when did you start getting headache
    a. 10 years and younger
    b. 25 years
    c. 35 years
    d. 45 years
    e. 50 years
22. how many day do you have headache per month
    a. less than 15 days
    b. Equal or more than 15 days/month
23. Did you have diagnosis for the headache?
    a. Yes
    b. no
24. What type of headache
    a. Tension type headache
    b. Migraine type
    c. Cluster headache
    d. other

25. Do you use headache over the counter analgesic ?

a. Yes
b. No
c. Sometime

38. if you take the analgesia during the attack what do you take

a. panadol, fevadol,pandrix (paracetamol group-nonopioid analgesics)
b. ibuprofen diclofenac naproxen (NSAID group- nonopioid analgesics)
c. solpadeine fevadol plus (paracetamol, codeine caffeine- opioids analgesics)
d. relaxon (paracetamol, muscle relaxant)
e. other

39. How long do you use the analgesic like paracetamol ibuprofen ergotamine triptan over the past year
a. One-2 times per week
b. 3 times per week
c. More than 4 times per week

40. For how long your using the analgesic on this pattern :
a. Less than 3 months
b. More than 3 months

41. How do you get the analgesic?
a. self-experience
b. advertisement flyer
c. pharmacist
d. family or friend
e. doctor

42. If you went to the doctor what specialty did you visit?
a. Family medicine
b. Neurology
c. Internal medicine

Preventive medicine for headache

43. did you try any medication as preventive medicine for headache
a. yes
b. no

44. If yes , what is it?
a. amitriptyline
b. topamax
c. gabapentin
d. lyrica
e. verapamil
f. botox
g. other:
h. I am not using any medicine

45. Do you still use the preventive medicine ?
a. Yes
b. No

46. if you stop use it why
    a. i had side effect from it
    b. i read the side effect in the pamphlet
    c. i am worry if might cause addiction
    d. my family/ friend advise me to stop it
    e. a doctor advises me to stop it
